# Covert Communications in a Hybrid DF/AF Relay System

**DOI:** 10.3390/s24206518

**Published:** 2024-10-10

**Authors:** Jihwan Moon

**Affiliations:** Department of Mobile Convergence Engineering, Hanbat National University, Daejeon 34158, Republic of Korea; anschino@staff.hanbat.ac.kr

**Keywords:** physical layer security, covert communications, low probability of detection, decode-and-forward, amplify-and-forward

## Abstract

In this paper, we study covert communications in a hybrid decode-and-forward (DF)/ amplify-and-forward (AF) relay system. The considered relay in normal operation forwards messages from a source node to a destination node in either DF or AF mode on request. Meanwhile, the source and destination nodes also attempt to secretly exchange covert messages such as confidential or sensitive information and avoid detection by the covert message detector embedded on the relay. We first establish an optimal DF/AF mode selection criterion to maximize the covert rate based on the analyses of delay-aware achievable covert rates of individual DF and AF modes. To further reduce the time complexity, we propose a low-complexity selection criterion as well for practical use. The numerical results demonstrate the covert rate gain as high as 50% and running time gain as high as 20% for particular system parameters, which verify the effectiveness of the proposed criteria.

## 1. Introduction

As wireless technology pervades our daily routines, security and privacy threats have increasingly become paramount concerns nowadays [1]. To ensure the security of wireless transmissions, covert communications, or low-probability-of-detection communications, has been considered as a promising physical layer security technology, which focuses on hiding the very existence of a confidential communications link [2]. It is a particularly useful technology for critical environments such as battlefields, security facilities, and financial institutions where the mere detection or metadata of data traffic could compromise the whole system [3].

The survey paper [4] has explored a vast number of works on covert communications, ranging from the fundamental principle to applications in various system categories in the existing literature. One of the remarkable works introduced is [5] on a three-node Gaussian channel topology composed of a transmitter, receiver, and warden. The authors revealed that the amount of bits that can be covertly transmitted is subject to a square root law and limited by $O(\sqrt{n})$ in *n* channel uses, which tends to 0 as n→∞. Their later work [6] explained that achieving a positive covert rate is possible in the presence of uncertainties in covert transmission, channels, and noise distribution. Another work [7] on the same three-node network showed that the Gaussian signaling is optimal for maximizing the covert rate with a lower bound on the minimum detection error probability (DEP) at the warden. The authors in the more recent work [8] further derived an optimal signaling with a peak power constraint, and [9] discussed potential applications of covert communications strategies including [8] to the sixth generation (6G) non-terrestrial network (NTN).

There also has been increasing interest in the potential of covert communications in relay systems. In [10], the authors minimized the average age of information (AoI) in a covert amplify-and-forward (AF) relay network by optimizing friendly artificial noise (AN) to a warden. The authors in [11] maximized the covert rate of unmanned aerial vehicle (UAV)-aided relay communications by optimizing the trajectory and phases of the intelligent reflecting surface (IRS) on the UAV. Also in [12], the authors jointly optimized the beamforming vectors of a transmitter and decode-and-forward (DF) relay in the presence of a multi-antenna warden when the relay can switch on and off to achieve the best covert rate. The authors in [13] derived optimal transmit power controls of a transmitter, selected DF relay, and cooperative jammers by taking the DEP and transmission outage probability due to random channel variables into consideration. In the presence of an active warden which emits fixed-power jamming signals, the authors of [14] derived optimal transmit power for a transmitter and selected DF relay. Both downlink and uplink IRS-aided non-orthogonal multiple access (NOMA) covert transmissions were considered in [15], and the authors maximized the effective covert rate by optimizing power allocation in Nakagami-*m* fading environments. For two-way IRS-based relay covert communications, the authors of [16] identified the maximum expected DEP subject to covert rate threshold by optimizing the cooperative AN power and covert transmission probability. In [17], the authors studied minimizing the expected relative entropy at the multi-antenna warden, which is equivalent to maximizing the DEP, by optimizing the source and DF relay transmit power in the presence of imperfect source and relay beamforming vectors. The authors in [18] investigated multi-user uplink covert transmissions through a multi-antenna relay that scrambles the received message before forwarding to a multi-antenna destination in order to break the correlation in the two phases of relay procedure, which may further confuse a multi-antenna warden. Furthermore, in [19], the authors jointly optimized the transmit power and trajectory of an UAV relay as well as the phase shifts of an IRS implemented on it for a clustered NOMA covert communications scenario.

Although there are numerous types of a relay device including an IRS, UAV, and mobile, relay protocols can generally be classified into three types: DF, compress-and-forward (CF), and AF. Our recent works in [20,21] identified and compared the optimal performance of the three relay protocols. The analytical and numerical results not only provided useful guidelines for practical operations but also suggested potential for further enhancement by an optimal relay mode selection. The authors of [22] studied combinations of half-duplex (HD)/full-duplex (FD) and DF/AF and identified the maximum covert rate with an external warden. However, the relay processing delay difference between DF and AF, which is an important factor for deciding the relay mode in practice, was not explicitly taken into account. In addition, there was no structured method to select the modes beside numerical comparisons. We thus build solid algorithms that choose the best relay mode for the maximum covert rate upon the foundational analyses from [20,21] with the relay processing delay.

Concretely, in this work, the considered relay in normal operation forwards messages from a source node to a destination node in either DF or AF mode on request. Meanwhile, the source and destination nodes also attempt to secretly exchange covert messages, e.g., confidential or sensitive information, and avoid detection by the covert message detector embedded on the relay. Our contributions for such a system are summarized as follows:

- We develop an efficient optimal DF/AF relay mode selection algorithm upon the analyses on the optimal covert rates of each relay mode. Compared to a naive method that calculates all related equations, this algorithm narrows down the required comparisons and performs only necessary calculations to reach the optimal mode selection.
- To further reduce the time complexity, we propose a low-complexity mode selection algorithm based on the long-term statistics of channel variables. It computes computationally expensive logarithms at the beginning of a long time block during which the wireless channel is statistically static. Then, it utilizes the stored results for relay mode selection in each time instant.
- We provide numerical results to highlight the covert communications performance gain of the hybrid DF/AF relay. The results also verify the correctness of the proposed algorithms and let us explore the effects of different system parameters on covert rates.

## 2. System Model

**Figure 1:**
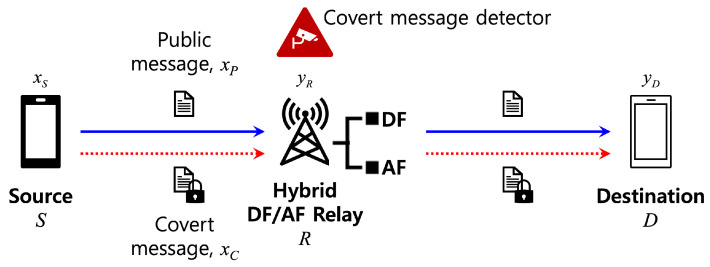
System model.

Figure 1 illustrates our considered hybrid DF/AF relay system with an embedded covert message detector. The source node *S* secretly transmits covert messages to the destination node *D* under cover of public messages. The relay *R* in normal operation forwards messages in either DF or AF mode based on the request from the source node. We assume that the *S*-*D* direct link is unusable due to shadowing or a far distance [14].

The relay receives

(1) $$\begin{array}{c} y_{R}=h_{SR}\sqrt{P_{S}}\left(\sqrt{\alpha }x_{P}+\sqrt{1-\alpha }x_{C}\right)+z_{R}, \end{array}$$

where $h_{\mathrm{XY}}$ specifies the channel coefficient from node X to Y for X,Y∈{S,R,D}, $x_{P}\;∼\;CN\left(0,1\right)$ and $x_{C}∼CN\left(0,1\right)$ mean the public and covert messages, respectively, $P_{S}$ denotes source transmit power, α indicates the proportion of $P_{S}$ on $x_{P}$, and $z_{R}∼CN\left(0,\sigma_{R}^{2}\right)$ represents the additive noise at the relay. We take into account the noise uncertainty at the relay [23,24]. That is, $\sigma_{R,\mathrm{dB}}^{2}∼U\left({\bar{\sigma }}_{R,\mathrm{dB}}^{2}-\zeta_{\mathrm{dB}},{\bar{\sigma }}_{R,\mathrm{dB}}^{2}+\zeta_{\mathrm{dB}}\right)$ in decibel scale, where ${\bar{\sigma }}_{R,\mathrm{dB}}^{2}$ and $\zeta_{\mathrm{dB}}\geq 0$ express the mean and range of noise power, respectively. Since covert communications is executed under the normal operation of relay, the global channel state information is assumed available everywhere [20,21].

Let us first review the DEP derivation from our previous work [20] based on the assumption that the covert message detector on the relay is equipped with a radiometer, which is a practical detection equipment [23,25]. We will also revisit the achievable covert rates of each DF and AF relay mode derived in [20] by considering the relay processing delay difference from [26] subsequently.

### 2.1. Covert Message Detection

The covert message detector at the relay in the considered system determines the existence of a covert transmission by measuring the level of extra components apart from the expected public message in the received signal. By examining such a residual signal ${\tilde{z}}_{R}≜y_{R}-h_{SR}\sqrt{P_{S}}x_{P}$ with the perfect knowledge of $h_{SR}$ and $P_{S}$ [27], we may write the null and alternative hypotheses as

(2) $$\begin{array}{c} \begin{array}{cc} H_{0}: & {\tilde{z}}_{R}=z_{R},\\ H_{1}: & {\tilde{z}}_{R}=h_{SR}\sqrt{P_{S}}\left(\left(\sqrt{\alpha }-1\right)x_{P}+\sqrt{1-\alpha }x_{C}\right)+z_{R}. \end{array} \end{array}$$

The null hypothesis $H_{0}$ and alternative hypothesis $H_{1}$ are formed upon the absence and presence of a covert message, respectively. The equipped radiometer then measures the average power *T* of the received signal as

(3) $$\begin{array}{c} \begin{array}{cc} H_{0}: & T=\sigma_{R}^{2},\\ H_{1}: & T=2{\left|h_{SR}\right|}^{2}P_{S}\left(1-\sqrt{\alpha }\right)+\sigma_{R}^{2}, \end{array} \end{array}$$

and the decision of the presence of a covert message is made upon T≥τ for some threshold τ.

The DEP Pr(e) consists of false alarm and miss probabilities in the form of

(4) $$\begin{array}{c} \mathrm{Pr}\left(e\right)=\underset{\mathrm{False}\;\mathrm{alarm}}{\underset{︸}{\mathrm{Pr}\left(T\geq \tau |H_{0}\right)}}\mathrm{Pr}\left(H_{0}\right)+\underset{\mathrm{Miss}}{\underset{︸}{\mathrm{Pr}\left(T<\tau |H_{1}\right)}}\mathrm{Pr}\left(H_{1}\right). \end{array}$$

We further consider a random covert transmission with $\mathrm{Pr}\left(H_{0}\right)=\mathrm{Pr}\left(H_{1}\right)=0.5$ [5]. Then, the optimal τ minimizing the DEP is given by [21]

(5) $$\begin{array}{c} \tau^{★}=2{\left|h_{SR}\right|}^{2}P_{S}\left(1-\sqrt{\alpha }\right)+\frac{1}{\zeta }{\bar{\sigma }}_{R}^{2}, \end{array}$$

whose minimum DEP results in [21]

(6) $$\begin{array}{c} {\left.\mathrm{Pr}\left(e\right)\right|}_{\tau =\tau^{★}}=\frac{1}{2}\left(1-\frac{1}{2\ln\zeta }\left(\ln\frac{\tau^{★}}{\tau^{★}-2{\left|h_{SR}\right|}^{2}P_{S}\left(1-\sqrt{\alpha }\right)}\right)\right), \end{array}$$

subject to $\zeta {\bar{\sigma }}_{R}^{2}\geq 2{\left|h_{SR}\right|}^{2}P_{S}\left(1-\sqrt{\alpha }\right)+{\bar{\sigma }}_{R}^{2}/\zeta$. This can be perceived as the worst-case minimum DEP on the assumption that the detector knows the exact value of α.

### 2.2. The Achievable Covert Rate in DF Relay Mode

Based on (1), the achievable rate of the *S*-*R* link is given by

(7) $$\begin{array}{c} {\bar{r}}_{S}=\log_{2}\left(1+\frac{{\left|h_{SR}\right|}^{2}P_{S}}{\sigma_{R}^{2}}\right). \end{array}$$

Then, the relay decodes and forwards the combined message $x_{S}≜\sqrt{\alpha }x_{P}+\sqrt{1-\alpha }x_{C}$ to the destination node, and the destination node receives

(8) $$\begin{array}{c} y_{D}=h_{RD}\sqrt{P_{R}}\left(\sqrt{\alpha }x_{P}+\sqrt{1-\alpha }x_{C}\right)+z_{D}, \end{array}$$

for the relay transmit power $P_{R}$ and additive noise $z_{D}∼CN\left(0,\sigma_{D}^{2}\right)$. The achievable rate of the *R*-*D* link is calculated as

(9) $$\begin{array}{c} {\bar{r}}_{R}=\log_{2}\left(1+\frac{{\left|h_{RD}\right|}^{2}P_{R}}{\sigma_{D}^{2}}\right). \end{array}$$

It should be noted that the actual data rate for $x_{S}$ must be less than or equal to (7) and (9) for successful decoding at the relay and destination node from information theory [28].

At the destination node, decoding $x_{P}$ precedes obtaining $x_{C}$. Concretely, taking $x_{C}$ part as interference first, the achievable rate for $x_{P}$ is written as [15]

(10) $$\begin{array}{c} r_{P,\mathrm{DF}}=\log_{2}\left(1+\frac{{\left|h_{RD}\right|}^{2}P_{R}\alpha }{{\left|h_{RD}\right|}^{2}P_{R}\left(1-\alpha \right)+\sigma_{D}^{2}}\right), \end{array}$$

and the achievable rate for $x_{C}$ after removing the decoded $x_{P}$ can be derived as

(11) $$\begin{array}{c} r_{C,\mathrm{DF}}=\log_{2}\left(1+\frac{{\left|h_{RD}\right|}^{2}P_{R}\left(1-\alpha \right)}{\sigma_{D}^{2}}\right). \end{array}$$

It is also worth noting that the actual data rates for $x_{P}$ and $x_{C}$ must be less than or equal to (10) and (11), respectively, for successful decoding.

With these in hand, Section 4.1 in our previous work [20] has derived the maximum achievable worst-case covert rate for DF relay mode subject to the minimum guaranteed public rate ${\bar{r}}_{P}$ and DEP ε∈[0,0.5] as

(12) $$\begin{array}{c} r_{C,\mathrm{DF}}^{★}=\min\left(\log_{2}\left(1+\gamma_{SR,\min}\right)-{\bar{r}}_{P},\log_{2}\left(1+\gamma_{RD}\right)-{\bar{r}}_{P},\log_{2}\left(1+\gamma_{RD}\left(1-\bar{\alpha }\right)\right)\right), \end{array}$$

where $\gamma_{SR,\min}≜{\left|h_{SR}\right|}^{2}P_{S}/\left(\zeta {\bar{\sigma }}_{R}^{2}\right)$, $\gamma_{RD}≜{\left|h_{RD}\right|}^{2}P_{R}/\sigma_{D}^{2}$, and 

(13) $$\begin{array}{c} \bar{\alpha }≜{\left(\max\left(1-\left(\zeta^{1-4\varepsilon }-\frac{1}{\zeta }\right)\frac{{\bar{\sigma }}_{R}^{2}}{2{\left|h_{SR}\right|}^{2}P_{S}},0\right)\right)}^{2}. \end{array}$$

### 2.3. The Achievable Covert Rate in AF Relay Mode

For AF relay mode, we can write the received signal at the destination node as

(14) $$\begin{array}{c} y_{D}=h_{RD}\sqrt{P_{R}}x_{R}+z_{D}, \end{array}$$

where $x_{R}≜y_{R}/{\left({\left|h_{SR}\right|}^{2}P_{S}+\sigma_{R}^{2}\right)}^{1/2}$ indicates the normalized forward signal. The achievable rates for $x_{P}$ and $x_{C}$ following the identical successive decoding in Section 2.2 are then given by

(15) $$\begin{array}{cc} \; & r_{P,\mathrm{AF}}=\log_{2}\left(1+\frac{\gamma_{SR}\alpha }{\gamma_{SR}\left(1-\alpha \right)+1+\left(\gamma_{SR}+1\right)\gamma_{RD}^{-1}}\right), \end{array}$$

(16) $$\begin{array}{cc} \; & r_{C,\mathrm{AF}}=\log_{2}\left(1+\frac{\gamma_{SR}\left(1-\alpha \right)}{1+\left(\gamma_{SR}+1\right)\gamma_{RD}^{-1}}\right), \end{array}$$

respectively, where $\gamma_{SR}≜{\left|h_{SR}\right|}^{2}P_{S}/\sigma_{R}^{2}$. The previous work [21] and Section 4.3 of [20] have also derived the maximum achievable worst-case covert rate for the AF relay mode subject to the minimum guaranteed public rate ${\bar{r}}_{P}$ and DEP ε∈[0,0.5] as

(17) $$\begin{array}{c} r_{C,\mathrm{AF}}^{★}=\min\left(r_{C,\mathrm{AF},1},r_{C,\mathrm{AF},2}\right), \end{array}$$

where

(18) $$\begin{array}{cc} \; & r_{C,\mathrm{AF},1}≜\log_{2}\left(1+\frac{\gamma_{SR,\min}}{1+\left(\gamma_{SR,\min}+1\right)\gamma_{RD}^{-1}}\right)-{\bar{r}}_{P}, \end{array}$$

(19) $$\begin{array}{cc} \; & r_{C,\mathrm{AF},2}≜\log_{2}\left(1+\frac{\gamma_{SR,\min}\left(1-\bar{\alpha }\right)}{1+\left(\gamma_{SR,\min}+1\right)\gamma_{RD}^{-1}}\right). \end{array}$$

### 2.4. Relay Processing Delay

The authors in [26] investigated the relationship between the total end-to-end packet transmission delays $t_{e2e,\mathrm{DF}}$ and $t_{e2e,\mathrm{AF}}$, and relay processing delays $t_{R,\mathrm{DF}}$ and $t_{R,\mathrm{AF}}$ for DF and AF modes, respectively. Specifically, if $t_{\mathrm{dec}}$ represents the decoding delay,

(20) $$\begin{array}{cc} t_{e2e,\mathrm{DF}} & =n+t_{R,\mathrm{DF}}+t_{\mathrm{dec}}=2\left(n+t_{\mathrm{dec}}\right), \end{array}$$

(21) $$\begin{array}{cc} t_{e2e,\mathrm{AF}} & =n+t_{R,\mathrm{AF}}+t_{\mathrm{dec}}=2n+t_{\mathrm{dec}}, \end{array}$$

for *n* channel uses per message where $t_{R,\mathrm{DF}}=n+t_{\mathrm{dec}}$ and $t_{R,\mathrm{AF}}=n$. We adopt a linear decoding delay in terms of *n* by $t_{\mathrm{dec}}=\delta n$ for some δ≥0, which is appropriate to embrace different coding schemes [29]. The delay factor δ quantifies the amount of difference in processing time between the modes. For instance, high δ means a large difference (Although δ∈[0,4] was chosen for simulation results in [26], it should be emphasized that δ in practice is specific to particular hardware configurations of interest and may be larger.).

Dividing $r_{C,\mathrm{DF}}^{★}$ by $t_{e2e,\mathrm{DF}}$ and $r_{C,\mathrm{AF}}^{★}$ by $t_{e2e,\mathrm{AF}}$ thus leads to delay-normalized achievable worst-case covert rates as

(22) $$\begin{array}{cc} r_{C,\mathrm{DF},\;\mathrm{delay}-\mathrm{norm}}^{★} & =\frac{r_{C,\mathrm{DF}}^{★}}{t_{e2e,\mathrm{DF}}}=\frac{1}{2\left(1+\delta \right)n}r_{C,\mathrm{DF}}^{★}, \end{array}$$

(23) $$\begin{array}{cc} r_{C,\mathrm{AF},\;\mathrm{delay}-\mathrm{norm}}^{★} & =\frac{r_{C,\mathrm{AF}}^{★}}{t_{e2e,\mathrm{AF}}}=\frac{1}{\left(2+\delta \right)n}r_{C,\mathrm{AF}}^{★}. \end{array}$$

Without loss of generality, we may focus on the number of transmitted bits for duration of $t_{e2e,\mathrm{AF}}$ as

(24) $$\begin{array}{cc} r_{C,\mathrm{DF},\;\mathrm{delay}}^{★} & =t_{e2e,\mathrm{AF}}r_{C,\mathrm{DF},\;\mathrm{delay}-\mathrm{norm}}^{★}=\frac{2+\delta }{2\left(1+\delta \right)}r_{C,\mathrm{DF}}^{★}, \end{array}$$

(25) $$\begin{array}{cc} r_{C,\mathrm{AF},\;\mathrm{delay}}^{★} & =t_{e2e,\mathrm{AF}}r_{C,\mathrm{AF},\;\mathrm{delay}-\mathrm{norm}}^{★}=r_{C,\mathrm{AF}}^{★}, \end{array}$$

by which we only need to adjust $r_{C,\mathrm{DF},\;\mathrm{delay}}^{★}$ for comparison. It is worth noting that the scaling equally applies to the public rate $r_{\mathrm{DF}}$ for DF mode, possibly resulting in a lower value than the minimum guaranteed public rate ${\bar{r}}_{P}$. To prevent this, we can first replace ${\bar{r}}_{P}$ of (P1) in [20] by the higher threshold ${\bar{r}}_{P}\left(2\left(1+\delta \right)\right)/\left(2+\delta \right)$ and scale both the obtained $r_{P,\mathrm{DF}}^{★}$ and $r_{C,\mathrm{DF}}^{★}$ by (2+δ)/(2(1+δ)) subsequently. Taking this into consideration, the final expression of the delay-aware achievable covert rate for DF mode is written by

(26) $$\begin{array}{c} r_{C,\mathrm{DF},\;\mathrm{delay}}^{★}=\min\left(r_{C,\mathrm{DF},\;\mathrm{delay},1},r_{C,\mathrm{DF},\;\mathrm{delay},2},r_{C,\mathrm{DF},\;\mathrm{delay},3}\right), \end{array}$$

with

(27) $$\begin{array}{cc} \; & r_{C,\mathrm{DF},\;\mathrm{delay},1}≜\frac{2+\delta }{2\left(1+\delta \right)}\log_{2}\left(1+\gamma_{SR,\min}\right)-{\bar{r}}_{P}, \end{array}$$

(28) $$\begin{array}{cc} \; & r_{C,\mathrm{DF},\;\mathrm{delay},2}≜\frac{2+\delta }{2\left(1+\delta \right)}\log_{2}\left(1+\gamma_{RD}\right)-{\bar{r}}_{P}, \end{array}$$

(29) $$\begin{array}{cc} \; & r_{C,\mathrm{DF},\;\mathrm{delay},3}≜\frac{2+\delta }{2\left(1+\delta \right)}\log_{2}\left(1+\gamma_{RD}\left(1-\bar{\alpha }\right)\right). \end{array}$$

## 3. Problem Formulation

In this work, we aim to establish an optimal mode selection criterion for the hybrid DF/AF relay to maximize the covert rate by solving

(30a) $$\begin{array}{cc} (P1):\; & \max_{a_{AF}}\;r_{C,\mathrm{DF},\;\mathrm{delay}}^{★}\left(1-a_{AF}\right)+r_{C,\mathrm{AF}}^{★}a_{AF}, \end{array}$$

(30b) $$\begin{array}{cc} \mathrm{subject}\;\mathrm{to}:\; & a_{AF}\in \left\{0,1\right\}, \end{array}$$

where $a_{AF}$ determines the relay mode. A naive approach to (P1) is to evaluate the covert rates of both DF mode from (26) and AF mode from (17) and select the higher one. This exhaustive search method, however, not only induces high time complexity but also prevents gaining insights into the solution. Hence, we will present a criterion that optimally selects the best mode without having to entirely calculate both (26) and (17). To further reduce the time complexity, we also provide a simpler criterion utilizing long-term statistics.

**Remark 1.**  *Let us take addition, subtraction or comparison as* Δ *running times, multiplication or division as* $\Delta^{2}$ *running times, exponentiation or logarithm as* $\Delta^{2}\log\Delta$ *running times for* Δ *bits of an input value based on [30]. We will count the running times of the exhaustive method for selecting the optimal relay mode.*

*In order to obtain (26) for DF and (17) for AF modes, the constants* $\gamma_{SR,\min}$*,* $\gamma_{RD}$ *and* $\bar{\alpha }$ *in (13) must to be calculated.*$\gamma_{SR,\min}$ *and* $\gamma_{RD}$ *require* 4 *and* 3 *multiplications or divisions, respectively, and *$\bar{\alpha }$ *in (13) takes* 2 *subtractions,* 7 *multiplications or divisions,* 1 *exponentiation, and* 1 *comparison from* max(·) *operation. This results in* $3\Delta +14\Delta^{2}+\Delta^{2}\log\Delta$ *running times.*

*Next, each calculation of* $r_{C,\mathrm{DF},\;\mathrm{delay},1}$ *in (27) and* $r_{C,\mathrm{DF},\;\mathrm{delay},2}$ *in (28) incurs* 4 *additions or subtractions,* 3 *multiplications or divisions, and* 1 *logarithm, while* $r_{C,\mathrm{DF},\;\mathrm{delay},3}$ *in (29) takes* 4 *additions or subtractions,* 4 *multiplications or divisions, and* 1 *logarithm. Therefore, including* 2 *comparisons among them to determine* $r_{C,\mathrm{DF},\;\mathrm{delay}}^{★}$ *from (26), the total running times become* $14\Delta +10\Delta^{2}+3\Delta^{2}\log\Delta$*.*

*As for AF mode,* $r_{C,\mathrm{AF},1}$* in (18) needs* 4 *additions or subtractions,* 2 *multiplications or divisions, and* 1 *logarithm. Meanwhile,* $r_{C,\mathrm{AF},2}$* in (19) takes* 4* additions or subtractions,* 3* multiplications or divisions, and* 1* logarithm. Including* 1* comparison among them to decide* $r_{C,\mathrm{AF}}^{★}$* from (17) thus leads to* $9\Delta +5\Delta^{2}+2\Delta^{2}\log\Delta$*.*

*Finally, after performing* 1* comparison between* $r_{C,\mathrm{DF},\;\mathrm{delay}}^{★}$* and* $r_{C,\mathrm{AF}}^{★}$*, we have* $27\Delta +29\Delta^{2}+6\Delta^{2}\log\Delta$* running times altogether.*

## 4. Proposed Solutions

### 4.1. An Optimal Criterion

For DF mode, if we define $\omega ≜\min(\gamma_{SR,\min},\gamma_{RD})$ and let $i_{\omega }=1$ if $\omega =\gamma_{SR,\min}$ and 2 if $\omega =\gamma_{RD}$, (26) becomes equivalent to

(31) $$\begin{array}{c} r_{C,\mathrm{DF},\;\mathrm{delay}}^{★}=\min\left(r_{C,\mathrm{DF},\;\mathrm{delay},i_{\omega }},r_{C,\mathrm{DF},\;\mathrm{delay},3}\right). \end{array}$$

The selection in min(·) depends on (27)–(29) such that

(32) $$\begin{array}{c} {\bar{r}}_{P,\mathrm{boundary},\mathrm{DF}}≜\frac{2+\delta }{2\left(1+\delta \right)}\log_{2}\left(\frac{1+\omega }{1+\gamma_{RD}\left(1-\bar{\alpha }\right)}\right)\overset{r_{C,\mathrm{DF},\;\mathrm{delay},i_{\omega }}}{\underset{r_{C,\mathrm{DF},\;\mathrm{delay},3}}{≶}}{\bar{r}}_{P}. \end{array}$$

**Figure 2:**
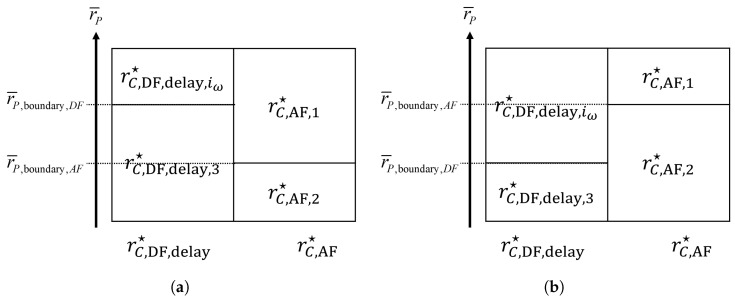
An optimal DF/AF mode selection criterion based on ${\bar{r}}_{P}$. (**a**) ${\bar{r}}_{P,\mathrm{boundary},\mathrm{DF}}\geq {\bar{r}}_{P,\mathrm{boundary},\mathrm{AF}}$. (**b**) ${\bar{r}}_{P,\mathrm{boundary},\mathrm{DF}}<{\bar{r}}_{P,\mathrm{boundary},\mathrm{AF}}$.

In AF mode, we can draw a similar selection rule for (17) from (18) and (19) as

(33) $$\begin{array}{c} {\bar{r}}_{P,\mathrm{boundary},\mathrm{AF}}≜\log_{2}\left(\frac{1+\lambda }{1+\lambda \left(1-\bar{\alpha }\right)}\right)\overset{r_{C,\mathrm{AF},1}}{\underset{r_{C,\mathrm{AF},2}}{≶}}{\bar{r}}_{P}, \end{array}$$

where $\lambda ≜\gamma_{SR,\min}/\left(1+\left(\gamma_{SR,\min}+1\right)\gamma_{RD}^{-1}\right)$. Therefore, an optimal DF/AF mode selection criterion differs depending on ${\bar{r}}_{P}$ with respect to ${\bar{r}}_{P,\mathrm{boundary},\mathrm{DF}}$ and ${\bar{r}}_{P,\mathrm{boundary},\mathrm{AF}}$ (Figure 2):

- Case 1: $\min\left({\bar{r}}_{P,\mathrm{boundary},\mathrm{DF}},{\bar{r}}_{P,\mathrm{boundary},\mathrm{AF}}\right)\geq {\bar{r}}_{P}$
  $r_{C,\mathrm{DF},\;\mathrm{delay}}^{★}=r_{C,\mathrm{DF},\;\mathrm{delay},3}$ and $r_{C,\mathrm{AF}}^{★}=r_{C,\mathrm{AF},2}$, i.e.,
  (34) $$\begin{array}{c} \frac{2+\delta }{2\left(1+\delta \right)}\log_{2}\left(1\;+\;\gamma_{RD}\left(1\;-\;\bar{\alpha }\right)\right)\overset{\mathrm{AF}}{\underset{\mathrm{DF}}{≶}}\log_{2}\left(1\;+\;\lambda \left(1\;-\;\bar{\alpha }\right)\right). \end{array}$$
- Case 2: ${\bar{r}}_{P,\mathrm{boundary},\mathrm{AF}}<{\bar{r}}_{P}\leq {\bar{r}}_{P,\mathrm{boundary},\mathrm{DF}}$
  $r_{C,\mathrm{DF},\;\mathrm{delay}}^{★}=r_{C,\mathrm{DF},\;\mathrm{delay},3}$ and $r_{C,\mathrm{AF}}^{★}=r_{C,\mathrm{AF},1}$, i.e.,
  (35) $$\begin{array}{c} \frac{2+\delta }{2\left(1+\delta \right)}\log_{2}\left(1+\gamma_{RD}\left(1-\bar{\alpha }\right)\right)\overset{\mathrm{AF}}{\underset{\mathrm{DF}}{≶}}\log_{2}\left(1+\lambda \right)-{\bar{r}}_{P}. \end{array}$$
- Case 3: ${\bar{r}}_{P,\mathrm{boundary},\mathrm{DF}}<{\bar{r}}_{P}\leq {\bar{r}}_{P,\mathrm{boundary},\mathrm{AF}}$
  $r_{C,\mathrm{DF},\;\mathrm{delay}}^{★}=r_{C,\mathrm{DF},\;\mathrm{delay},i_{\omega }}$ and $r_{C,\mathrm{AF}}^{★}=r_{C,\mathrm{AF},2}$, i.e.,
  (36) $$\begin{array}{c} \frac{2+\delta }{2\left(1+\delta \right)}\log_{2}\left(1+\omega \right)-{\bar{r}}_{P}\overset{\mathrm{AF}}{\underset{\mathrm{DF}}{≶}}\log_{2}\left(1+\lambda \left(1-\bar{\alpha }\right)\right). \end{array}$$
- Case 4: $\max\left({\bar{r}}_{P,\mathrm{boundary},\mathrm{DF}},{\bar{r}}_{P,\mathrm{boundary},\mathrm{AF}}\right)<{\bar{r}}_{P}$
  $r_{C,\mathrm{DF},\;\mathrm{delay}}^{★}=r_{C,\mathrm{DF},\;\mathrm{delay},i_{\omega }}$ and $r_{C,\mathrm{AF}}^{★}=r_{C,\mathrm{AF},1}$, i.e.,
  (37) $$\begin{array}{c} \frac{2+\delta }{2\left(1+\delta \right)}\log_{2}\left(1+\omega \right)\overset{\mathrm{AF}}{\underset{\mathrm{DF}}{≶}}\log_{2}\left(1+\lambda \right). \end{array}$$

Algorithm 1 summarizes the selection procedure between DF and AF in four steps.
**Algorithm 1** The proposed optimal mode selection.1:Deciding ω and $i_{\omega }$ for DF mode by computing and comparing $\gamma_{SR,\min}$ and $\gamma_{RD}$2:Deciding between $r_{C,\mathrm{DF},\;\mathrm{delay},i_{\omega }}$ or $r_{C,\mathrm{DF},\;\mathrm{delay},3}$ as $r_{C,\mathrm{DF},\;\mathrm{delay}}^{★}$ by comparing ${\bar{r}}_{P,\mathrm{boundary},\mathrm{DF}}$ and ${\bar{r}}_{P}$ after computing $\bar{\alpha }$ and ${\bar{r}}_{P,\mathrm{boundary},\mathrm{DF}}$3:Deciding between $r_{C,\mathrm{AF},1}$ or $r_{C,\mathrm{AF},2}$ as $r_{C,\mathrm{AF}}^{★}$ by comparing ${\bar{r}}_{P,\mathrm{boundary},\mathrm{AF}}$ and ${\bar{r}}_{P}$ after computing λ and ${\bar{r}}_{P,\mathrm{boundary},\mathrm{AF}}$4:Deciding the relay mode by computing and comparing $r_{C,\mathrm{DF},\;\mathrm{delay}}^{★}$ and $r_{C,\mathrm{AF}}^{★}$

**Remark 2.**  *We can evaluate the running times of Algorithm 1 based on the similar assumption in Remark 1. Step 1 requires calculation of $\gamma_{SR,\min}$ and $\gamma_{RD}$ with *4* and *3* multiplications or divisions, respectively, and *1* comparison for deciding $i_{\omega }$, summing up to $\Delta +7\Delta^{2}$ running times.*

*In step 2, calculation of $\bar{\alpha }$ in (13) takes *2* subtractions, *7* multiplications or divisions, *1* exponentiation, and *1* comparison, which add up to $3\Delta +7\Delta^{2}+\Delta^{2}\log\Delta$ running times. Computing ${\bar{r}}_{P,\mathrm{boundary},\mathrm{DF}}$ consumes *5* additions or subtractions, *5* multiplications or divisions, and *1* logarithm, leading to $5\Delta +5\Delta^{2}+\Delta^{2}\log\Delta$ running times. After that, the comparison between ${\bar{r}}_{P,\mathrm{boundary},\mathrm{DF}}$ and ${\bar{r}}_{P}$ for deciding $r_{C,\mathrm{DF}}^{★}$ adds *Δ* running times. This is $8\Delta +12\Delta^{2}+2\Delta^{2}\log\Delta$ running steps in total.*

*In step 3, obtaining λ requires *2* additions, *2* divisions for $2\Delta +2\Delta^{2}$ running times. ${\bar{r}}_{P,\mathrm{boundary},\mathrm{AF}}$ also requires *3* additions or subtractions, *2* multiplications or divisions, and *1* logarithm for $3\Delta +2\Delta^{2}+\Delta^{2}\log\Delta$ running times. Then, the comparison between ${\bar{r}}_{P,\mathrm{boundary},\mathrm{AF}}$ and ${\bar{r}}_{P}$ for determining $r_{C,\mathrm{AF}}^{★}$ adds *Δ* running times such that the total complexity becomes $6\Delta +4\Delta^{2}+\Delta^{2}\log\Delta$.*

*For step 4, evaluating $r_{C,\mathrm{DF},\;\mathrm{delay}}^{★}$ needs *4* additions or subtractions, *4* multiplications or divisions at most, and *1* logarithm, which total $4\Delta +4\Delta^{2}+\Delta^{2}\log\Delta$ running times. Finding $r_{C,\mathrm{AF}}^{★}$ takes *4* additions or subtractions, *3* multiplications or divisions at most, and *1* logarithm that amount to $4\Delta +3\Delta^{2}+\Delta^{2}\log\Delta$ running times. Including *1* comparison between $r_{C,\mathrm{DF},\;\mathrm{delay}}^{★}$ and $r_{C,\mathrm{AF}}^{★}$ to select the final relay mode, the total running times become $9\Delta +7\Delta^{2}+2\Delta^{2}\log\Delta$.*

*Altogether, Algorithm 1 takes $24\Delta +30\Delta^{2}+5\Delta^{2}\log\Delta$. Since exponentiation and logarithm with $\Delta^{2}\log\Delta$ running times are the major factors for computational complexity, we can conclude that Algorithm 1 has lower complexity compared to the exhaustive search method from Remark 1.*

### 4.2. A Low-Complexity Partial Long-Term Criterion

The proposed optimal mode selection criterion requires calculation of several parameters that vary with instantaneous channel state information. A straightforward method to decrease the computational burden at each time instant would be utilizing approximated ${\tilde{r}}_{C,\mathrm{DF},\;\mathrm{delay}}^{★}$ and ${\tilde{r}}_{C,\mathrm{AF}}^{★}$ upon the long-term statistics $E[{\left|h_{SR}\right|}^{2}]$ and $E[{\left|h_{RD}\right|}^{2}]$ at the cost of inaccurate selection.

Thus, we propose a low-complexity algorithm that takes an adequate trade-off between the accuracy and time complexity by making partial use of the long-term statistics. To this end, we first note that the last step in Algorithm 1 involves evaluating logarithms, which are generally expensive compared to simple arithmetic operators and take up the most computational resource. We thus retain the use of instantaneous channel coefficients in steps 1–3 but replace them with the long-term counterparts in step 4. This enables the system to evaluate ${\tilde{r}}_{C,\mathrm{DF},\;\mathrm{delay}}^{★}$ and ${\tilde{r}}_{C,\mathrm{AF}}^{★}$ and store the results of (34)–(37) prior to the beginning of each time block. The relay mode will then be instantly selected after step 3. The modified procedure is described in Algorithm 2.
**Algorithm 2** The proposed low-complexity partial long-term mode selection.1:Evaluating ${\tilde{r}}_{C,\mathrm{DF},\;\mathrm{delay}}^{★}$ and ${\tilde{r}}_{C,\mathrm{AF}}^{★}$, and storing the results of (24)–(27) in the beginning of each time block2:Deciding ω and $i_{\omega }$ for DF mode by computing and comparing $\gamma_{SR,\min}$ and $\gamma_{RD}$3:Deciding between $r_{C,\mathrm{DF},\;\mathrm{delay},i_{\omega }}$ or $r_{C,\mathrm{DF},\;\mathrm{delay},3}$ as $r_{C,\mathrm{DF},\;\mathrm{delay}}^{★}$ by comparing ${\bar{r}}_{P,\mathrm{boundary},\mathrm{DF}}$ and ${\bar{r}}_{P}$ after computing $\bar{\alpha }$ and ${\bar{r}}_{P,\mathrm{boundary},\mathrm{DF}}$4:Deciding between $r_{C,\mathrm{AF},1}$ or $r_{C,\mathrm{AF},2}$ as $r_{C,\mathrm{AF}}^{★}$ by comparing ${\bar{r}}_{P,\mathrm{boundary},\mathrm{AF}}$ and ${\bar{r}}_{P}$ after computing λ and ${\bar{r}}_{P,\mathrm{boundary},\mathrm{AF}}$5:Deciding the relay mode based on step 1

**Remark 3.**  *Assuming the one-time calculations of ${\tilde{r}}_{C,\mathrm{DF},\;\mathrm{delay}}^{★}$ and ${\tilde{r}}_{C,\mathrm{AF}}^{★}$ per transmission block to be negligible in step 1, we may only count the running times for step 2, 3, and 4 of Algorithm 2, which are equivalent to step 1, 2, and 3 of Algorithm 2, respectively. The comparison in step 5 can also be precalculated based on step 1. Therefore, the effective total running times for Algorithm 2 is $15\Delta +23\Delta^{2}+3\Delta^{2}\log\Delta$.*

We summarize the complexity comparison in Table 1. It can be found that the running times for Δ→∞ by all the algorithms are dominated by the logarithm term $\Delta^{2}\log\Delta$. The asymptotic decrements in the running time by Algorithms 1 and 2 over the exhaustive search are thus 16% and 50%, respectively.

## 5. Numerical Results

We now present numerical simulations to assess the covert communications performance of our proposed algorithms. We place the source, hybrid DF/AF relay, and destination nodes on a straight line. The distance-dependent channel coefficient $h_{\mathrm{XY}}$ from node X to Y for X,Y∈{S,R,D} is in the form of $h_{\mathrm{XY}}=\sqrt{L_{\mathrm{XY}}}{\hat{h}}_{\mathrm{XY}}$ in which $L_{\mathrm{XY}}≜L_{0}{\left(d_{\mathrm{XY}}/d_{0}\right)}^{-\beta }$ and ${\hat{h}}_{\mathrm{XY}}$ denote the path loss and small-scale channel variation, respectively [31]. Here, $L_{0}$ indicates the path loss at a reference distance $d_{0}=1$ m, β accounts for the path loss exponent, and ${\hat{h}}_{\mathrm{XY}}$ is assumed to follow Rayleigh fading with CN(0,1).

We set other system parameters as follows: the bandwidth W=20 MHz, *R*-*S* distance $d_{RS}=100$ m, *R*-*D* distance $d_{RD}=100$ m, source transmission power $P_{S}=23$ dBm, relay transmission power $P_{R}=23$ dBm, mean noise power at the relay ${\bar{\sigma }}_{R}^{2}=-160$ dBm/Hz, noise uncertainty range ζ=5 dB, destination noise power $\sigma_{D}^{2}=-160$ dBm/Hz, DEP threshold ε=0.45, pathloss exponent β=3.5, public rate threshold ${\bar{r}}_{P}=1.5$ bps/Hz, and processing delay factor δ=5.0.

**Figure 3:**
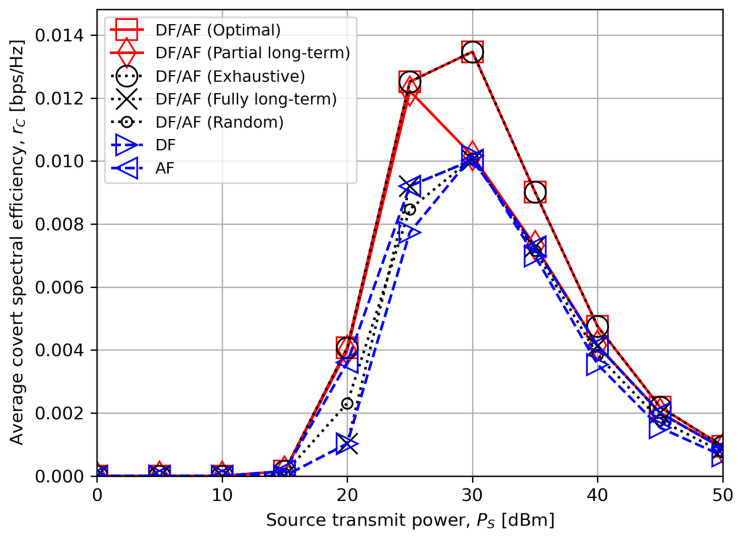
The average worst-case covert rate $r_{C}$ versus the source transmit power $P_{S}$.

Figure 3 shows the average worst-case covert rate as a function of the source transmit power $P_{S}$. We observe that the optimal mode selection of the hybrid DF/AF relay (“DF/AF (Optimal)”) yields a significant performance gain over the single relay modes (“DF” and “AF”). The equivalence between “DF/AF (Optimal)” and “DF/AF (Exhaustive)” in which the best relay mode is selected after evaluating all the logarithms in (18) and (19), (27)–(29) also verifies the correctness of our proposed criterion. The performance gain of the optimal criterion over the other compared schemes is approximately 35% at $P_{S}=30$ dBm.

The figure confirms the effectiveness of our proposed low-complexity algorithm (“DF/AF (Partial long-term)”) as well. It is found that the performance is close to the optimum for low $P_{S}$ values, but deviates from the optimum for higher $P_{S}$. Nevertheless, the low-complexity scheme still outperforms other baseline schemes such as “DF/AF (Fully long-term)” where the selection is determined solely by the long-term statistics of channel, and “DF/AF (Random)” with random selection.

**Figure 4:**
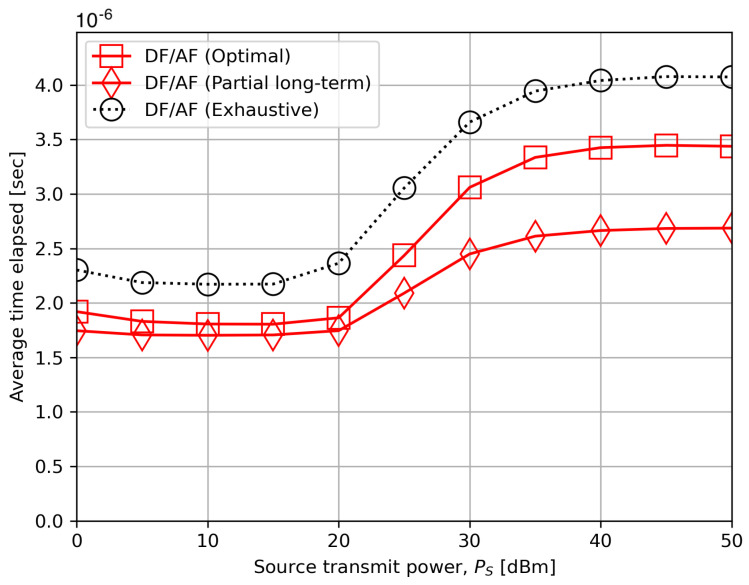
The average time elapsed versus the source transmit power $P_{S}$.

Figure 4 compares the average time elapsed among “DF/AF (Optimal)”, “DF/AF (Partial long-term)”, and “DF/AF (Exhaustive)” when $P_{S}$ increases. We conducted this experiment using Python 3.10 on 2023 MacBook Air 15 (Apple Inc., Cupertino, CA, USA) with Apple M2 processor including 4 cores of 3.49 GHz and the other 4 cores of 2.42 GHz. Both the proposed schemes have achieved notably lower time consumption compared to the exhaustive method. For instance, the running time gains of the optimal and partial long-term criteria over the exhaustive search are approximately 15% and 34%, respectively, at $P_{S}=40$ dBm, and 21% and 26% at $P_{S}=20$ dBm. We can thus conclude from Figure 3 and Figure 4 that the proposed schemes provide a satisfactory trade-off between performance and time complexity.

**Figure 5:**
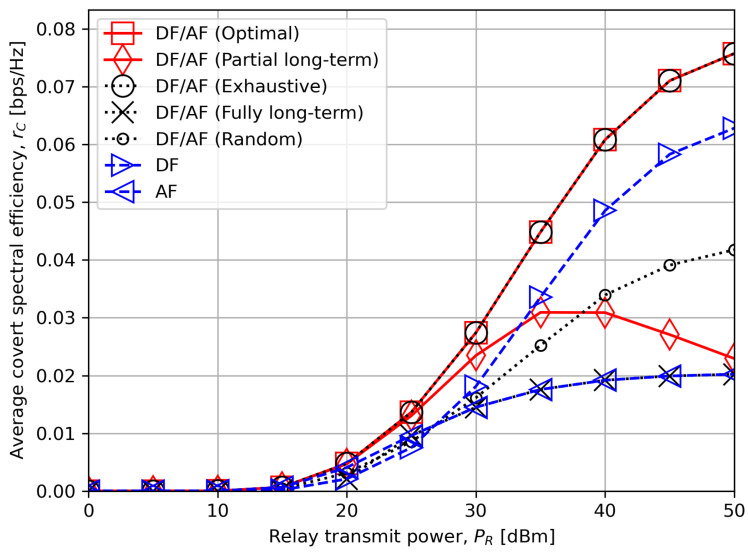
The average worst-case covert rate $r_{C}$ versus the relay transmit power $P_{R}$.

Figure 5 studies the relationship between the average worst-case covert rate and relay transmit power $P_{R}$. The optimal hybrid DF/AF scheme once again validates its effectiveness for all $P_{R}$ values. As an example, the performance improvement of the optimal criterion over the compared schemes is at least approximately 20% at $P_{S}=40$ dBm. The low-complexity algorithm also demonstrates greater performance over other compared schemes from low to medium $P_{R}$ regimes, but it reduces to the single AF relay mode as $P_{R}$ becomes high. In fact, when $P_{R}$ is high, $\gamma_{RD}$ is also high, and this results in $r_{C,\mathrm{DF},\;\mathrm{delay}}^{★}\to r_{C,\mathrm{DF},\;\mathrm{delay},1}$ from (26) and (27), and $\omega \to \gamma_{SR,\min}$ and $\lambda \to \gamma_{SR,\min}$ by definition. Moreover, one can numerically check that both average ${\bar{r}}_{P,\mathrm{boundary},\mathrm{DF}}$ and ${\bar{r}}_{P,\mathrm{boundary},\mathrm{AF}}$ are much lower than ${\bar{r}}_{P}=1.5$ bps/Hz for our choice of system parameters in this section. This corresponds to Case 4 in (37) in which AF is highly likely to be selected since ω≃λ for high $P_{R}$ and δ=5.0. Hence, it explains why “DF/AF (Partial long-term)” scheme approaches the single AF relay mode. A more careful selection is therefore necessary for high $P_{R}$.

**Figure 6:**
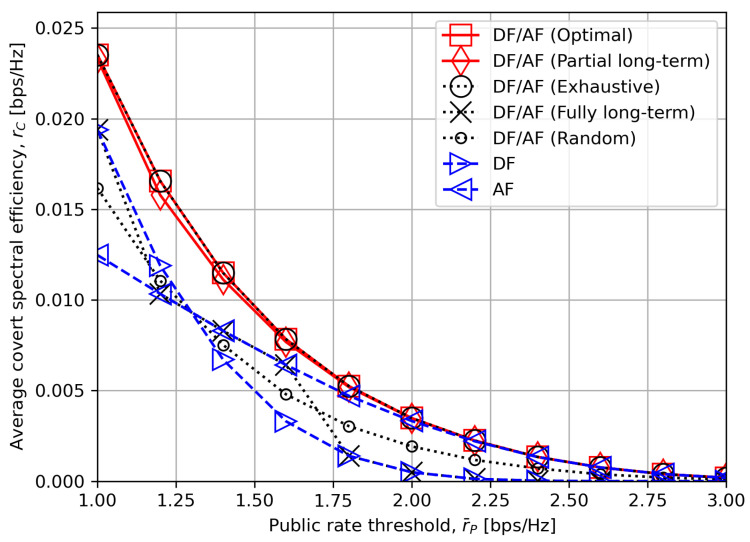
The average worst-case covert rate $r_{C}$ versus the public rate threshold ${\bar{r}}_{P}$.

Figure 6 presents the average worst-case covert rate as the public rate threshold ${\bar{r}}_{P}$ varies. Our proposed optimal and low-complexity mode selection schemes show considerable improvement than other compared ones for all ${\bar{r}}_{P}$ ranges. For ${\bar{r}}_{P}=1.3$ as an example, the optimal criterion gains 28% improvement at least over the other compared schemes. We note that “DF/AF (Fully long-term)” based only on the long-term statistics of channel even underperform the random mode selection, despite its negligible computational complexity. This highlights the decent trade-off between the complexity and performance by the proposed “DF/AF (Partial long-term)”.

It also appears that AF outperforms DF as ${\bar{r}}_{P}$ becomes higher. This is due to the stricter requirement for decodability at both the relay and destination node for DF mode while it is only the destination node that needs to perform decoding for AF mode. For instance, ${\bar{r}}_{P}$ is compared with $\gamma_{SR,\min}$ and $\gamma_{RD}$ in both $r_{C,\mathrm{DF},\;\mathrm{delay},1}$ in (27) and $r_{C,\mathrm{DF},\;\mathrm{delay},2}$ in (28) for DF mode. On the other hand, it is only $r_{C,\mathrm{AF},1}$ in (18) that is compared with ${\bar{r}}_{P}$ for AF mode. Thus, higher ${\bar{r}}_{P}$ is unfavorable for DF mode to simultaneously meet the decodability at both the relay and destination node.

**Figure 7:**
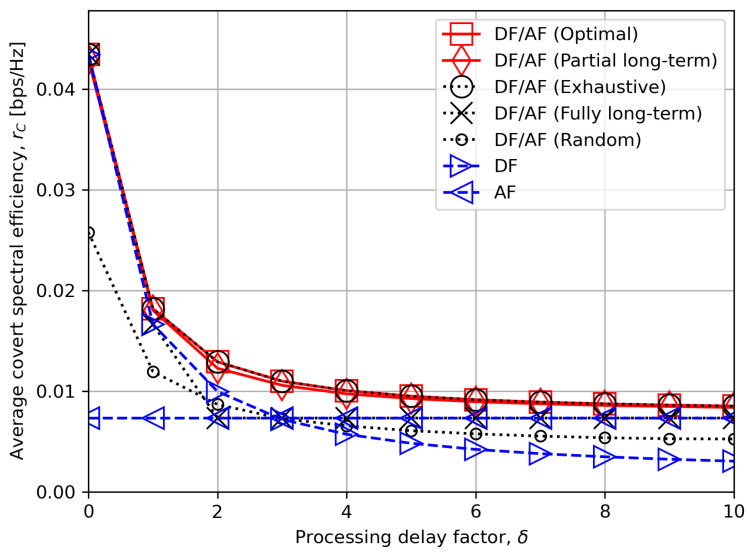
The average worst-case covert rate $r_{C}$ versus the processing delay factor δ.

Figure 7 illustrates the average worst-case covert rate performance for different values of processing delay factor δ. The proposed optimal and low-complexity mode selection schemes mostly choose DF when δ is low and AF when δ is high as expected. Besides, both schemes make the best decision when δ is in an intermediate range. The performance gain of 50% is observed for the optimal criterion over the other compared schemes for δ=3.0. In contrast, “DF/AF (Fully long-term)” only with the long-term statistics of channel was not able to determine the best relay mode for non-extreme values of δ, which could be more common in practice.

## 6. Conclusions

In this paper, we studied covert communications in a hybrid DF/AF relay system. The considered relay in normal operation forwards messages from a source node to a destination node in either DF or AF mode on request. Meanwhile, the source and destination nodes also attempt to secretly exchange covert messages, e.g., confidential or sensitive information, and avoid detection by the covert message detector embedded on the relay. We first established an optimal DF/AF mode selection criterion to maximize the covert rate based on the analyses of delay-aware achievable covert rates of individual DF and AF modes. To further reduce the time complexity, we proposed a low-complexity selection criterion as well for practical use. The numerical results demonstrate the covert rate gain as high as 50% and running time gain as high as 20% for particular system parameters, which verify the effectiveness of the proposed criteria.

The proposed hybrid DF/AF relay strategy for covert communications may provide adaptive and flexible means of secure transmissions by switching between DF and AF modes based on network conditions, security needs, and environmental threats, in favor of high covert rate. It is suitable for a wide range of real-world applications, such as a reliable and fast exchange of mission-critical information among military units in active combat zones, adaptive satellite-to-ground secure communications robust to abrupt climate changes and channel variations, and covert integrated sensing and communications with rapidly varying node locations.

An actual implementation of the proposed system model and relay mode selection algorithms, and handling related issues can be interesting future works.

## Figures and Tables

**Table 1:** The complexity comparison.

Algorithm	Running Time
Exhaustive search	$27\Delta +29\Delta^{2}+6\Delta^{2}\log\Delta$
Algorithm 1 (optimal)	$24\Delta +30\Delta^{2}+5\Delta^{2}\log\Delta$
Algorithm 2 (partial long-term)	$15\Delta +23\Delta^{2}+3\Delta^{2}\log\Delta$

Δ: The input bits of an input value.

## Data Availability

The data presented in this study are available on request from the corresponding author.

## References

[1] Zhang J., Yan Z., Fei S., Wang M., Li T., Wang H. (2022). Is Today’s End-to-End Communication Security Enough for 5G and Its Beyond?. IEEE Netw..

[2] Jiang Y., Wang L., Chen H.-H., Shen X. (2024). Physical Layer Covert Communication in B5G Wireless Networks—Its Research, Applications, and Challenges. Proc. IEEE.

[3] Forouzan B.A. (2007). Cryptography and Network Security.

[4] Chen X., An J., Xiong Z., Xing C., Zhao N., Yu F.R., Nallanathan A. (2023). Covert Communications: A Comprehensive Survey. IEEE Commun. Surv. Tutor..

[5] Bash B.A., Goeckel D., Towsley D. (2013). Limits of Reliable Communication with Low Probability of Detection on AWGN Channels. IEEE J. Sel. Areas Commun..

[6] Bash B.A., Goeckel D., Towsley D., Guha S. (2015). Hiding information in noise: Fundamental limits of covert wireless communication. IEEE Commun. Mag..

[7] Yan S., Cong Y., Hanly S.V., Zhou X. (2019). Gaussian Signalling for Covert Communications. IEEE Trans. Wirel. Commun..

[8] Kang B., Ye N., An J. (2024). Optimal Signaling for Covert Communications Under Peak Power Constraint. IEEE Trans. Inf. Forensics Secur..

[9] An J., Kang B., Ouyang Q., Pan J., Ye N. (2024). Covert Communications Meet 6G NTN: A Comprehensive Enabler for Safety-Critical IoT. IEEE Netw..

[10] Sim S., Kim J., Lee J. Jamming Power Optimization for Data Freshness in Covert Relay Networks. Proceedings of the 2023 14th International Conference on Information and Communication Technology Convergence (ICTC).

[11] Qian Y., Yang C., Mei Z., Zhou X., Shi L., Li J. (2023). On Joint Optimization of Trajectory and Phase Shift for IRS-UAV Assisted Covert Communication Systems. IEEE Trans. Veh. Technol..

[12] Wang M., Xia B., Xu Z., Guo Y., Chen Z. (2023). Performance Analysis and Optimization for Coordinated Direct and Relay Covert Transmission With Multiantenna Warder. IEEE Internet Things J..

[13] Gao C., Yang B., Zheng D., Jiang X., Taleb T. (2024). Cooperative Jamming and Relay Selection for Covert Communications in Wireless Relay Systems. IEEE Trans. Commun..

[14] Zhao Q., Gao C., Zheng D., Li Y., Zheng X. (2024). Covert Communication in a Multirelay-Assisted Wireless Network With an Active Warden. IEEE Internet Things J..

[15] Li Q., Xu D., Zhang K., Navaie K., Ding Z. (2024). Covert Communications in STAR-RIS Assisted NOMA IoT Networks Over Nakagami-m Fading Channels. IEEE Internet Things J..

[16] Wang C., Xiong Z., Zheng M., Zhao N., Niyato D. (2024). Covert Communications via Two-Way IRS with Noise Power Uncertainty. IEEE Trans. Commun..

[17] Lin M., Liu C., Wu Q., Wang W. (2024). DF-Relay-Assisted Multiantenna D2D Covert Communication in the Presence of Joint Detection. IEEE Internet Things J..

[18] Lin M., Liu C., Wang W. (2024). Relay-Assisted Uplink Covert Communication in the Presence of Multi-Antenna Warden and Uninformed Jamming. IEEE Trans. Commun..

[19] Qin X., Na Z., Wen Z., Wu X. (2024). Relaying IRS-UAV Assisted Covert Communications in Uplink C-NOMA Network. IEEE Commun. Lett..

[20] Moon J. (2023). Performance Comparison of Relay-Based Covert Communications: DF, CF and AF. Sensors.

[21] Moon J. (2024). Covert Communications in a Compress-and-Forward Relay System. ICT Express.

[22] Liu Y., Wu H., Jiang X. (2023). Joint selection of FD/HD and AF/DF for covert communication in two-hop relay systems. Ad Hoc Netw..

[23] He B., Yan S., Zhou X., Lau V.K.N. (2017). On Covert Communication With Noise Uncertainty. IEEE Commun. Lett..

[24] Si J., Li Z., Zhao Y., Cheng J., Guan L., Shi J., Al-Dhahir N. (2021). Covert Transmission Assisted by Intelligent Reflecting Surface. IEEE Trans. Commun..

[25] Lee S., Baxley R.J., Weitnauer M.A., Walkenhorst B. (2015). Achieving Undetectable Communication. IEEE J. Sel. Top. Signal Process..

[26] Makki B., Alouini M.-S. (2019). End-to-End Performance Analysis of Delay-Sensitive Multi-Relay Networks. IEEE Commun. Lett..

[27] Kim S.W., Ta H.Q. (2022). Covert Communications Over Multiple Overt Channels. IEEE Trans. Commun..

[28] Cover T.M., Thomas J.A. (2005). Elements of Information Theory.

[29] Makki B., Svensson T., Caire G., Zorzi M. (2019). Fast HARQ Over Finite Blocklength Codes: A Technique for Low-Latency Reliable Communication. IEEE Trans. Wirel. Commun..

[30] Borwein J.M., Borwein P.B. (1987). Pi and the AGM: A Study in Analytic Number Theory and Computational Complexity.

[31] Xing H., Liu L., Zhang R. (2016). Secrecy Wireless Information and Power Transfer in Fading Wiretap Channel. IEEE Trans. Veh. Technol..

